# Supplementary figures and images for: Co-Expression of ZmVPP1 with ZmNAC111 Confers Robust Drought Resistance in Maize
Source: Genes (Basel). 2022 Dec 20;14(1):8. doi: 10.3390/genes14010008 (PMC9858277; doi:10.3390/genes14010008)

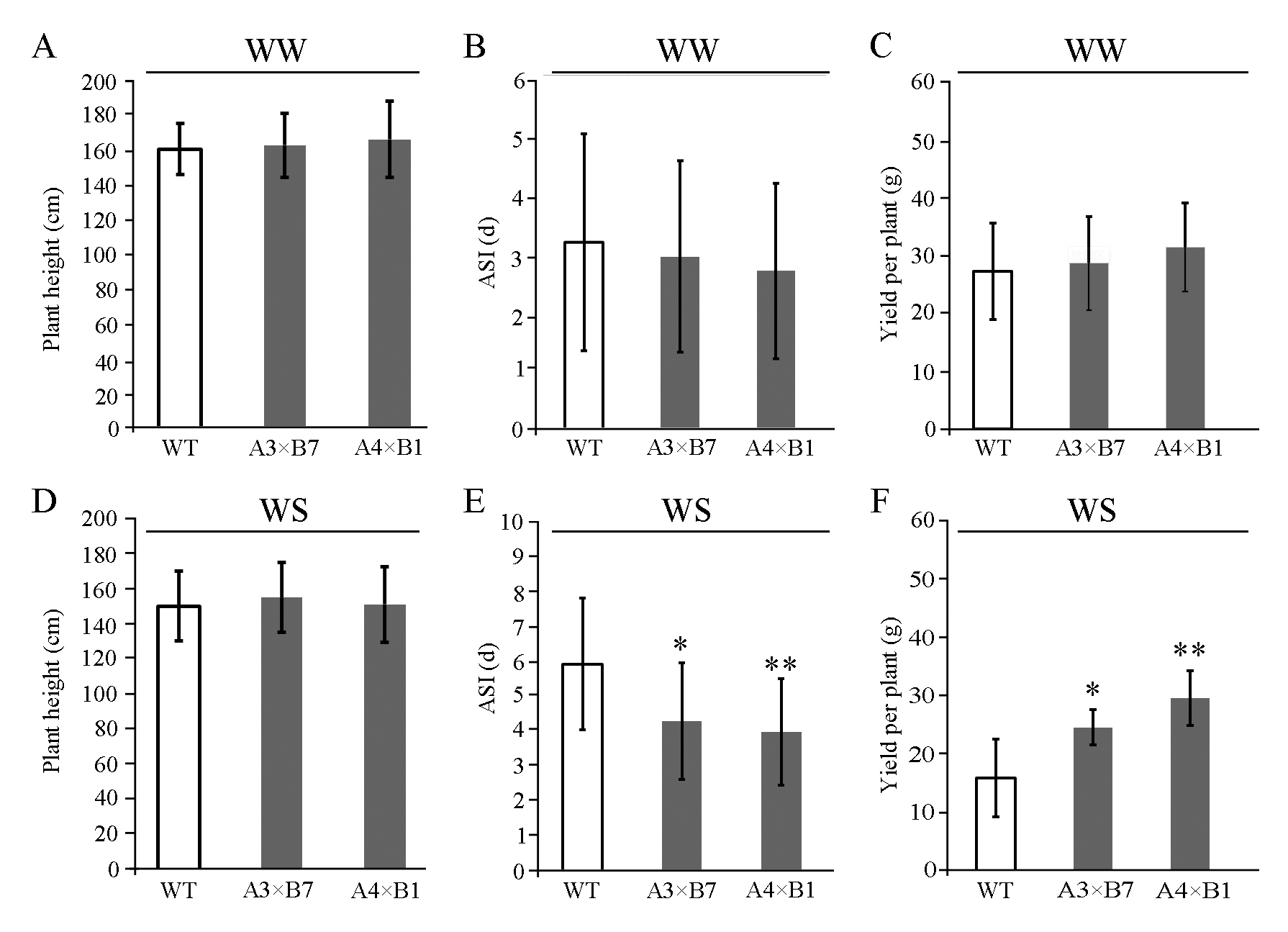

Supplement: Supplementary file 1 [file genes-14-00008-s001.zip › FigureS1.jpg]
